# Oncological and Reproductive Outcomes of a Standardized Hysteroscopic Approach for the Fertility-Sparing Treatment of Atypical Endometrial Hyperplasia and Early-Stage Endometrial Cancer

**DOI:** 10.3390/cancers18050839

**Published:** 2026-03-05

**Authors:** Ursula Catena, Emma Bonetti Palermo, Francesca De Bonis, Giulia Micol Bruni, Michela Zorzi, Eleonora La Fera, Giorgia Dinoi, Giacomo Corrado, Valeria Masciullo, Anna Fagotti, Francesco Fanfani

**Affiliations:** 1 Department of Woman and Child Health and Public Health, Fondazione Policlinico Universitario A. Gemelli, IRCCS, 00168 Rome, Italy; 2 Division of Obstetrics and Gynecology, Dipartimento Area Della Donna e Materno Infantile, ASST Spedali Civili Brescia, 25136 Brescia, Italy; 3 Department of Obstetrics and Gynecology, “San Giovanni di Dio” Hospital, 04022 Fondi, Italy; 4 Unit of Gynecology and Obstetrics, Department of Women’s and Children’s Health, University of Padova, 35141 Padova, Italy; 5 Department of Life Sciences and Public Health, Catholic University of the Sacred Heart, 00168 Rome, Italy

**Keywords:** fertility-sparing treatment (FST), hysteroscopy, atypical endometrial hyperplasia (AEH), endometrial cancer (EC), reproductive outcomes, oncological outcomes, progestin therapy, mini-resectoscope, tissue removal device (TRD)

## Abstract

Background/Objectives: Fertility-sparing treatment (FST) is an accepted option for selected women with atypical endometrial hyperplasia (AEH) and early-stage endometrioid endometrial cancer (EC). While combined progestin therapy and hysteroscopic approaches yield the best outcomes, the surgical component has long lacked standardization. This study aimed to evaluate oncological and reproductive outcomes following a standardized hysteroscopic fertility-sparing approach. Methods: This retrospective single-center study included women of reproductive age diagnosed with AEH or grade 1–2 endometrioid EC between 2021 and 2024 at the Digital Hysteroscopic Clinic CLASS Hysteroscopy, Fondazione Policlinico A. Gemelli IRCCS, Rome. All patients were treated using a standardized hysteroscopic approach based on lesion type and disease extension, combined with progestin therapy. Oncological outcomes included complete response (CR), time to CR and recurrence. Reproductive outcomes included the clinical pregnancy rate (CPR), live birth rate (LBR), and miscarriage rate (MR). Multivariate analysis was performed to identify factors associated with CR and relapse. Results: A total of 138 patients were included (79 AEH, 59 EC). CR rates were high and comparable between AEH (94.9%) and EC (98.2%), with a median time to response of 6 months. Recurrence rates did not differ significantly between groups (16.7% AEH vs. 26.8% EC). CPR was similar (47.5% AEH vs. 54.8% EC), with LBR exceeding 50% among women who conceived. Multivariate analysis identified age ≥ 35 years as the only factor associated with reduced response and increased relapse risk. Conclusions: A standardized hysteroscopic fertility-sparing approach combined with progestin therapy provides excellent oncologic control and favorable reproductive outcomes. Lesion severity did not affect outcomes when hysteroscopic removal was adequately performed, while patient age remains a key determinant of outcomes.

## Simple Summary

Fertility-sparing treatment (FST) is increasingly offered to young women with atypical endometrial hyperplasia (AEH) and early-stage endometrial cancer (EC) who wish to preserve their reproductive potential. However, the surgical approach to the conservative management of these patients has historically lacked standardization. In this study, we evaluated oncological and reproductive outcomes of women who underwent tailored hysteroscopic treatment, combined with progestin therapy. We found high rates of disease regression and favorable pregnancy outcomes in both AEH and early-stage EC, without significant differences between groups. Age emerged as the main factor influencing treatment response and recurrence, whereas lesion severity did not affect outcomes when hysteroscopic removal was adequately performed. These findings support the central role of adequate and standardized hysteroscopic surgery as a key element of FST and highlight its potential to preserve fertility without compromising oncologic safety when applied in specialized centers.

## 1. Introduction

Atypical endometrial hyperplasia (AEH) and early-stage endometrial cancer (EC) are increasingly diagnosed in women of reproductive age, largely driven by rising obesity rates and delayed childbearing [1]. Although EC predominantly affects postmenopausal women, approximately 25% of cases are diagnosed in premenopausal women, of whom 5–7% are younger than 40 years and the majority are nulliparous [2]. In this setting, the standard treatment—total hysterectomy with bilateral salpingo-oophorectomy and surgical staging—effectively eliminates the risk of disease progression but irreversibly impairs reproductive potential [3,4].

According to the ESGO/ESHRE/ESGE guidelines, fertility-sparing treatment (FST) is considered an acceptable alternative for carefully selected women with AEH and early-stage EC without myometrial invasion or extrauterine disease [5].

The recommended conservative approach combining high-dose progestin therapy with hysteroscopic tumor resection has been associated with the highest complete response and live birth rates if compared with progestin treatment alone [6]. However, the surgical approach of FST has remained heterogeneous for many years, without a universally standardized protocol. To address this gap, Catena et al. recently proposed a practical clinical flowchart built on current guidelines, defining a standardized surgical hysteroscopic management based on lesion type (AEH vs. focal EC vs. diffuse EC) and tumor grade [7].

The present study aimed to evaluate oncological and reproductive outcomes following this standardized approach.

## 2. Materials and Methods

### 2.1. Study Design and Population

This retrospective study was conducted between January 2021 and December 2024 at the Digital Hysteroscopic Clinic CLASS Hysteroscopy, Fondazione Policlinico A. Gemelli IRCCS in Rome. We included all consecutive women of reproductive age who underwent FST for AEH, and focal or diffuse grade 1–2 (G1-G2) endometrioid EC. Patients were excluded if they had evidence of myometrial invasion or extrauterine disease on imaging, non-endometrioid histology, or declined conservative management. All patients completed a standardized pre-treatment evaluation including endometrial biopsy under direct hysteroscopic visualization, transvaginal ultrasound and/or pelvic Magnetic Resonance Imaging (MRI) to confirm the absence of myometrial invasion, cervical extension, adnexal involvement and to exclude extrauterine disease. Pelvic MRI was performed when transvaginal ultrasound findings were inconclusive or when a more accurate assessment of myometrial invasion was required.

### 2.2. Surgical Standardized Technique and Progestin Therapy

All hysteroscopic procedures were performed by experienced hysteroscopic surgeons, using a vaginoscopic approach, without cervical dilation, under general anesthesia, following an ambulatory model of care [8]. The choice of surgical approach was based on the histological diagnosis and on the extent of endometrial disease at diagnostic hysteroscopy, in accordance with the fertility-sparing flowchart by Catena et al. [7].

**Figure 1:**
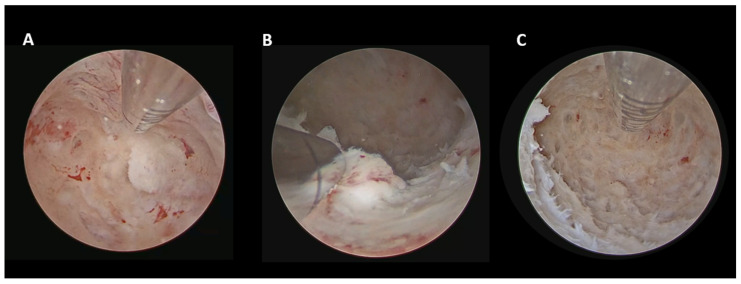
AEH: visual D&C with TRD. (**A**) Initial hysteroscopic view of the uterine cavity before treatment. (**B**) Intraoperative image showing the TRD in action. (**C**) Final view of the cavity after complete removal of hyperplastic tissue.

In cases of AEH (Figure 1), hysteroscopic management consisted of a so-called “visual dilation and curettage (D&C)” performed using a hysteroscopic tissue removal device (TRD), as described by Casadio et al. [9].

**Figure 2:**
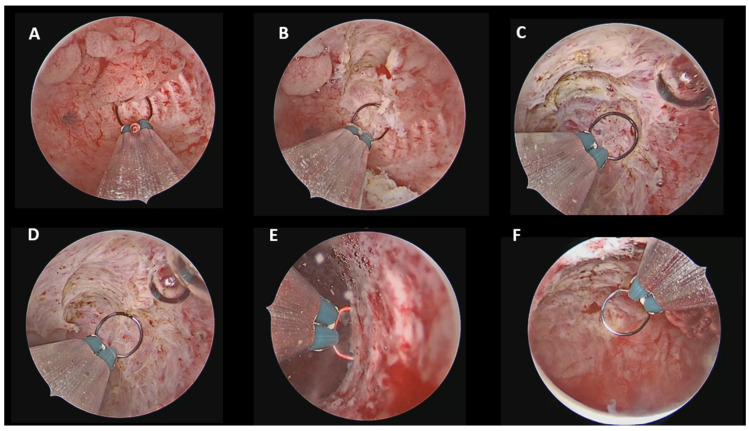
Focal EC: Mazzon “three-step” technique. (**A**) Initial hysteroscopic view of the focal lesion. (**B**) First step: excision of the lesion. (**C**) Second step: resection of the lateral endometrium (4–5 mm margins). (**D**) Third step: removal of the underlying myometrium (3–4 mm depth). (**E**) Random biopsies of the contralateral walls. (**F**) Final inspection showing complete removal and restored uterine cavity.

For focal endometrioid EC (Figure 2), the hysteroscopic resection was performed using a 15-Fr bipolar miniresectoscope following the established “three-step” technique described by Mazzon et al., consisting of complete excision of the visible lesion, resection of the surrounding endometrium approximately 4–5 mm beyond the lesion borders, and resection of the underlying myometrium to a depth of 3–4 mm [10].

**Figure 3:**
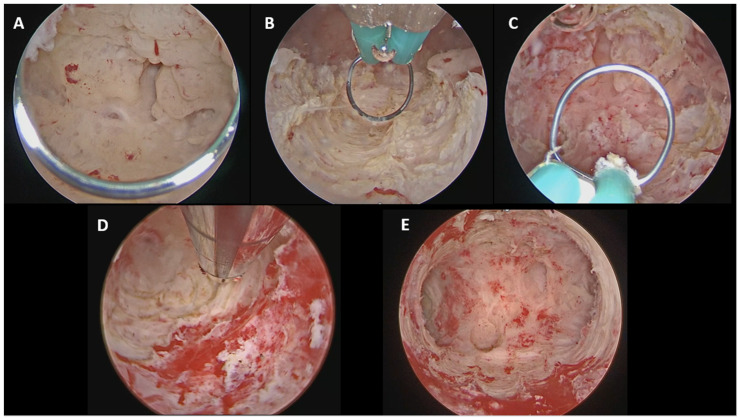
Diffuse EC: combined hysteroscopic treatment, according to Catena et al. (**A**) Initial hysteroscopic view of the diffuse endometrial cancer. (**B**) Resection of the main lesions with 15 Fr mini-resectoscope, reaching the myometrial plane. (**C**) Residual pathological tissue in difficult areas, such as tubal ostia. (**D**) Resection of the residual pathological tissue near tubal ostia with TRD. (**E**) Final view of the uterine cavity.

For diffuse endometrioid EC (Figure 3), a combined hysteroscopic technique was employed as detailed by Catena et al. [11]. A 15-Fr bipolar miniresectoscope was used to remove the main endometrial lesions according to the three-step principle reaching the myometrial plane, while a TRD was subsequently used to complete the resection in areas difficult to access, such as the tubal ostia.

Immediately after hysteroscopic lesion resection, all patients started progestin therapy. Treatment consisted of a levonorgestrel-releasing intrauterine device (LNG-IUD, 52 mg) and/or oral megestrol acetate (160 mg daily). A combined regimen with LNG-IUD plus oral megestrol acetate was preferentially adopted in patients with grade 2 EC or in cases of persistent or recurrent disease, to improve the complete remission rate.

### 2.3. Oncological and Reproductive Outcomes

Patients were followed up with scheduled office endometrial biopsy under direct hysteroscopic visualization, to evaluate response to FST. Complete response (CR) was defined as two consecutive negative endometrial biopsies, with a minimum interval of 3 months. The expected time to achieve CR was 6–12 months of treatment, in accordance with current recommendations [7,12]. Recurrence was defined as histologic evidence of AEH and/or EC after a documented CR.

Reproductive outcomes were evaluated in patients who achieved CR and discontinued progestin therapy to attempt to conceive. The clinical pregnancy rate (CPR), miscarriage rate (MR), and live birth rate (LBR) were assessed. CPR was defined as any pregnancy confirmed by ultrasound with visualization of a gestational sac and fetal heartbeat. MR was defined as the proportion of clinical pregnancies ending before 24 weeks of gestation. LBR was defined as the delivery of a live infant beyond 24 weeks of gestation. Mode of conception, spontaneous vs. assisted reproductive technology (ART), and mode of delivery were registered.

If pregnancy was not achieved, a 6-month office endometrial biopsy under direct hysteroscopic visualization was performed. Definitive surgery was recommended after completion of childbearing and in cases of disease progression or recurrence, and consisted of total hysterectomy with bilateral salpingo-oophorectomy and surgical staging; in selected patients with a persistent desire to preserve fertility, a second course of FST was considered. All data were retrieved from medical records.

### 2.4. Statistical Analysis

Categorical variables were reported as absolute numbers and frequency (percentages). Continuous variables were presented as median and interquartile range (IQR).

Comparisons between categorical variables were performed using the Chi-square test or Fisher’s exact test, as appropriate. Continuous variables were compared using the Mann–Whitney U test.

Multivariate analysis was conducted through a binomial logistic regression to establish the simultaneous effects of the independent variables in terms of odds ratios (ORs) on the oncological outcomes. Model calibration was assessed using the Hosmer–Lemeshow goodness-of-fit test.

All statistical analyses were performed using IBM SPSS Statistics software (version 26; IBM Corp., Armonk, NY, USA). A *p*-value < 0.05 was considered statistically significant for all tests.

### 2.5. Ethical Approval

The study adhered to the ethical principles outlined in the Declaration of Helsinki. The study was approved by the regional Medical Ethics Review Committee (Comitato Etico Territoriale Lazio Area 3) under reference number ID 7309 and registered on ClinicalTrials.gov (ID NCT07077876). Written informed consent was obtained from all participants.

## 3. Results

### 3.1. Patient Characteristics

Table 1 summarizes the baseline characteristics of the patients included in the study, stratified according to histological diagnosis (AEH vs. EC).

A total of 138 patients were included, of whom 79 (57.2%) were diagnosed with AEH and 59 (42.8%) with EC; among EC cases, 35 (59.3%) were grade 1 (G1) and 24 (40.7%) were grade 2 (G2).

The median age at diagnosis for the overall population was 36.0 years (IQR 31–40), with AEH patients being significantly older than those with EC (37.0 years (IQR 32–40) AEH vs. 34.0 years (IQR 29–38) EC, *p* = 0.01). Overall, more than half of the patients were older than 35 years (78/138, 56.5%); in particular, age ≥ 40 years was more frequent among AEH patients compared with EC patients (27/79, 34.2% AEH vs. 9/59, 15.3% EC, *p* = 0.01).

Median body mass index (BMI) also differed significantly between groups, being lower in AEH compared with EC (23.4 kg/m^2^ (IQR 20.9–29.4) AEH vs. 25.7 kg/m^2^ (IQR 23.2–30.8) EC, *p* = 0.03). Overall, more than half of the patients with available BMI data were at least overweight (BMI ≥ 25 kg/m^2^; 68/132, 51.5%), with a higher prevalence in the EC group compared with AEH (63.2% EC vs. 42.7% AEH, *p* = 0.02); one quarter of patients were obese (BMI ≥ 30 kg/m^2^; 34/132, 25.8%).

Regarding pre-diagnosis reproductive history, only 18 out of 138 patients (13.0%) had achieved pregnancies, with no significant difference between AEH and EC (12.7% AEH vs. 13.6% EC, *p* = 0.88). A history of miscarriages was reported in 12.3% patients (11.4% AEH vs. 13.6% EC, *p* = 0.70). No significant difference was found between the two groups in embryo transfer failure (1.3% AEH vs. 5.1% EC, *p* = 0.21).

Disease extension at hysteroscopic evaluation was more frequently focal in AEH compared with EC (54/79, 68.4% AEH vs. 30/59, 50.8% EC, *p* = 0.04), with diffuse involvement more common among EC patients (25/79, 31.6% AEH vs. 29/59, 49.2% EC, *p* = 0.04).

Progestin therapy differed significantly according to histological diagnosis (*p* < 0.01): overall, 87 patients (63.1%) received LNG-IUD alone, 9 (6.5%) oral progestins alone, and 42 (30.4%) combined therapy. Among AEH patients, LNG-IUD alone was the most common approach (68/79, 86.1%), whereas combined therapy was predominantly used in EC patients (36/59, 61.0%), including all grade 2 cases at baseline.

### 3.2. Oncological Outcomes

Table 2 summarizes the oncological outcomes of patients undergoing FST, divided according to histological diagnosis. For this analysis, 78 patients with AEH and 56 patients with EC were included, after exclusion of patients lost to follow-up (*n* = 1 AEH and *n* = 3 EC).

The CR rate was high in both groups, achieved in 74 of 78 patients with AEH (94.9%) and in 55 of 56 patients with EC (98.2%), without a statistically significant difference between groups (*p* = 0.30). One patient in the EC group showed a partial response. The median time to achieve CR was comparable between groups, being 6 months (range 6–12) in both AEH and EC patients (*p* = 0.42).

During follow-up, disease relapse occurred in 13 of 78 patients with AEH (16.7%) and in 15 of 56 patients with EC (26.8%), with no statistically significant difference between groups (*p* = 0.16). In the AEH group, relapses occurred both after progestin therapy discontinuation for pregnancy attempt (*n* = 8) or during ongoing progestin treatment (*n* = 5). Similarly, most relapses in the EC group were observed after progestin therapy discontinuation for pregnancy attempt (*n* = 13), while only 2 relapses occurred during ongoing treatment. Among relapsing EC cases, 8 were grade 2 and 7 were grade 1, with one grade 1 relapse showed histologic downgrading to AEH.

### 3.3. Reproductive Outcomes

Table 3 summarizes the reproductive outcomes of patients who attempted pregnancy after achieving CR and discontinuation of progestin therapy, stratified by AEH and EC. Reproductive outcomes were evaluated in 40 of 78 patients with AEH and in 31 of 56 patients with EC. Among patients with AEH, 38 were excluded from reproductive outcome analysis: 2 were lost to follow-up, 32 were still under progestin therapy, and 4 underwent radical surgery. Similarly, 25 patients with EC were excluded: 3 were lost to follow-up, 18 were still under progestin therapy, and 4 underwent radical surgery.

The median follow-up duration was 28 months (IQR 20–38).

The CPR was 47.5% (19/40) in the AEH group and 54.8% (17/31) in the EC group. Among patients who achieved a clinical pregnancy, the LBR was 57.9% (11/19) in AEH and 70.6% (12/17) in the EC. MR occurred in 15.8% (3/19) of AEH pregnancies and 17.6% (3/17) of EC pregnancies. At the time of analysis, ongoing pregnancies accounted for 26.3% (5/19) of pregnancies in the AEH group and 11.8% (2/17) in the EC group. ART was used to attempt pregnancy in 30.0% (12/40) of AEH patients and in 25.8% (8/31) of EC patients.

### 3.4. Multivariate Analysis of Oncological Outcomes

Two multivariate analyses were performed to identify factors associated with CR at 6 months and disease relapse (Table 4), respectively. Five clinically relevant variables were included in the model as independent variables: age ≥ 35 years, BMI ≥ 25 kg/m^2^, histological diagnosis (EC vs. AEH), disease extension (diffuse vs. focal), and tumor grade (grade 2 vs. grade 1-AEH).

Regarding CR, age ≥ 35 years was the only variable significantly associated with a reduced probability of achieving CR (OR 0.37, 95% CI 0.15–0.90; *p* = 0.03). BMI ≥ 25 kg/m^2^, histological diagnosis of EC, diffuse disease extension, and grade 2 tumors were not significantly associated with CR rates.

Regarding disease relapse, age ≥ 35 years also emerged as the only variable significantly associated with an increased risk of recurrence (OR 3.80, 95% CI 1.34–10.74; *p* = 0.01). None of the other variables included in the model showed a statistically significant association with relapse risk.

A sensitivity analysis restricted to patients within the common age overlap between groups (22–44 years; *n* = 72 AEH and *n* = 57 EC) confirmed the robustness of the findings, with age remaining the only predictor of CR and relapse.

Model calibration assessed using the Hosmer–Lemeshow goodness-of-fit test showed adequate fit for both models (*p* = 0.85 for CR; *p* = 0.57 for relapse).

## 4. Discussion

This single-center retrospective study supports the effectiveness of a standardized hysteroscopic surgical approach combined with progestin therapy for fertility-sparing management of AEH and early-stage endometrioid EC, highlighting the importance of a structured and reproducible treatment pathway.

Firstly, all candidates for FST of our cohort underwent a standardized pre-treatment workup based on endometrial biopsy under direct hysteroscopic visualization, performed in an office setting using 5 Fr hysteroscopic grasping forceps, which allows adequate amount of tissue for reliable histologic evaluation and grading [13,14]. Furthermore, all patients underwent preoperative imaging (transvaginal ultrasound and/or pelvic MRI) to exclude myometrial invasion and extrauterine disease [15]. Patients were counselled that radical surgery remains the standard treatment whereas FST represents a non-standard option requiring strict surveillance and definitive surgery after completion of childbearing [16].

In our cohort, relevant differences in patient characteristics were observed between AEH and EC patients, with age and BMI emerging as the main distinguishing factors. Although the median age of the overall population was relatively young (36.0 years), more than half of the patients were older than 35 years and nearly one quarter were aged 40 years or older, indicating that FST was frequently pursued in women approaching the upper limits of reproductive age. In particular, AEH patients were older than EC patients (median age 37.0 years vs. 34.0 years, *p* = 0.01), with the difference mainly driven by a higher proportion of women aged ≥ 40 years in the AEH group (34.2% AEH vs. 15.3% EC, *p* = 0.01). In contrast, BMI values were significantly higher among EC patients (median BMI 25.7 kg/m^2^ EC vs. 23.4 kg/m^2^ AEH, *p* = 0.03), with more than half of the EC group being at least overweight and approximately one quarter obese. These findings suggest that AEH may be associated with delayed diagnosis and prolonged exposure to non-obesity-related endocrine risk factors, such as insulin resistance, polycystic ovary syndrome, and chronic anovulation, whereas obesity appears to be more strongly associated with the development of more advanced pathology, such as EC [17,18,19].

Progestin therapy was chosen according to histological diagnosis and tumor grade, with LNG-IUD alone predominantly used in AEH (86.1%) and combined oral (Megestrol acetate 160 mg/day) plus LNG-IUD adopted in the majority of EC patients (61.0%), including all grade 2 cases at baseline, in line with evidence suggesting that combined progestin therapy may improve CR rates in grade 2 endometrioid EC [20].

The main strength of this study is the use of a fully standardized hysteroscopic surgical approach, where lesion removal represents a key component of FST. Beyond progestin therapy alone, complete and targeted hysteroscopic excision is a fundamental step in achieving adequate intrauterine cytoreduction while preserving reproductive potential [6,21,22,23,24,25,26]. In our clinical practice, surgical management was systematically guided by histology and disease extension, with a different approach for AEH and focal or diffuse EC. In our cohort, hysteroscopic findings revealed a higher prevalence of focal disease in AEH (68.4% AEH vs. 50.8% EC, *p* = 0.04) and a more frequent diffuse involvement in EC (31.6% AEH vs. 49.2% EC, *p* = 0.04). These findings support the pivotal role of diagnostic hysteroscopic assessment of the uterine cavity in surgical decision-making and the need for tailored surgical strategies according to disease extension.

According to our standardized hysteroscopic protocol, visual D&C using a TRD was adopted for AEH [9], focal hysteroscopic resection with a 15-Fr bipolar miniresectoscope for focal EC [10], and a combined approach (15-Fr bipolar miniresectoscope + TRD) for diffuse EC [11]. The choice of these instruments was driven by their reduced caliber and the possibility of performing the procedures in a minimally invasive manner without the need for cervical dilation.

The 15-Fr bipolar miniresectoscope allowed precise lesion targeting and controlled depth of resection for focal lesions, while also enabling effective coagulation when required. Its reduced caliber offers excellent maneuverability within the uterine cavity and minimizes the risk of cervical trauma compared with standard 26-Fr resectoscopes [27].

The use of TRD, based on a purely mechanical mechanism, enabled uniform and atraumatic endometrial removal without thermal damage. This feature is particularly advantageous in diffuse disease, as it reduces endometrial trauma and the risk of postoperative intrauterine adhesions while preserving endometrial integrity. Moreover, it helps in the resection of pathological tissue in anatomically challenging areas, such as near the tubal ostia, where the use of conventional electrosurgery may be technically demanding and dangerous [28,29]. Available evidence indicates that the use of TRD does not increase the risk of malignant cell dissemination, compared with other endometrial sampling techniques [30].

Our findings demonstrate excellent oncologic outcomes in both AEH and early-stage endometrioid EC, with high and comparable CR between groups (94.9% AEH vs. 98.2 EC, *p* = 0.30), and a similar median time to response of 6 months (range 6–12). These results are consistent with growing evidence supporting the combination of hysteroscopic tumor resection and progestin therapy as the most effective fertility-sparing strategy [5], as reported also by Giampaolino et al. who observed CR rates of 92.7% in AEH and 78.6% in early EC treated with hysteroscopic resection followed by LNG-IUD insertion, with low recurrence rates compared with progestin therapy alone [6]. Persistence of disease was observed in a small number of patients (4 with AEH and 1 with EC), possibly reflecting the heterogeneous hormonal background often characterizing premalignant conditions. Although relapse rates were numerically higher in EC group compared with AEH (26.8% EC vs. 16.7% AEH), this difference was not statistically significant (*p* = 0.16), suggesting that, when complete hysteroscopic resection is achieved, CR rate and recurrence risk may be comparable across different disease severities. Furthermore, most recurrences occurred after progestin therapy discontinuation for pregnancy attempt, highlighting the dynamic balance between oncological safety and reproductive goals and underscoring the importance of close surveillance during treatment discontinuation for pregnancy attempt.

In our cohort, reproductive outcomes were encouraging in both AEH and EC patients. Nearly half of the patients who attempted to conceive achieved a clinical pregnancy (47.5% AEH vs. 54.8% EC). Among women who conceived, LBR exceeded 50% in both groups (57.9% AEH vs. 70.6% EC). A substantial proportion of patients attempted pregnancy using ART (30.0% AEH and 25.8% EC), reflecting both underlying causes of subfertility and the clinical strategy of minimizing time to conception after treatment completion. Moreover, ongoing pregnancies were observed at the time of analysis in both groups (26.3% in AEH and 11.8% in EC), reinforcing the reproductive potential preserved by a standardized hysteroscopic fertility-sparing approach.

From our multivariate analysis, age ≥ 35 years emerged as the only variable associated with both a reduced probability of achieving CR within the first 6 months and an increased risk of relapse. These findings indicate that, when complete and standardized hysteroscopic lesion removal is achieved, the intrinsic severity of the disease—EC vs. AEH, diffuse vs. focal, grade 2 vs. lower grade—does not compromise CR rate or recurrence risk. Rather, patient-related factors, particularly age, appear to play a more relevant role in the effectiveness and durability of FST.

Limitations of this study include its retrospective design and single-center setting. In addition, molecular and immunohistochemical data were not systematically available and so were not included in the analysis. Immunohistochemistry, including mismatch repair (MMR) status, p53 expression and estrogen and progesterone receptors (ER and PR), was performed in fewer than half of the patients and only in those with endometrial carcinoma. Therefore, the prognostic impact of molecular alterations could not be evaluated. Additional limitations include the relatively limited follow-up for some patients treated in the most recent years and the absence of a control group treated with alternative fertility-sparing strategies.

Future research should focus on prospective, multicenter studies with longer follow-up to validate the oncological and reproductive outcomes observed in this series and to better define the long-term effectiveness of standardized hysteroscopic fertility-sparing protocols. The integration of molecular and immunohistochemical profiling, including MMR status, p53 expression, ER and PR, and POLE mutation analysis, will be essential to improve patient selection and risk stratification in future studies. Indeed, growing evidence suggests that molecular and immunohistochemical classification may significantly influence response and recurrence rates after FST, with MMR-deficient and p53-abnormal tumors showing less favorable oncologic outcomes compared with other molecular subtypes [31,32,33,34,35]. In addition, future studies should explore the impact of metabolic and endocrine comorbidities, particularly in women with AEH, such as insulin resistance and polycystic ovary syndrome, which are often associated with amenorrhea and chronic anovulation and may negatively influence reproductive outcomes, potentially increasing the need for ART. Multicenter and prospective studies are needed to clarify these associations and their clinical implications.

## 5. Conclusions

This study supports the effectiveness of a standardized hysteroscopic fertility-sparing approach combined with progestin therapy for AEH and early-stage endometrioid EC. When hysteroscopic lesion removal is performed in a structured and reproducible manner according to histology and disease extension, high CR rates and acceptable relapse rates can be achieved, independently of lesion severity.

Age emerged as the main determinant of response and relapse, whereas histological diagnosis, grade, and disease extension did not affect outcomes, highlighting the central role of adequate surgical intrauterine cytoreduction. These findings emphasize the importance of patient selection, a standardized hysteroscopic technique, and close follow-up, while future prospective and multicenter studies integrating molecular profiling are needed to refine risk stratification and optimize personalized fertility-sparing strategies.

## Figures and Tables

**Table 1:** Characteristics of the 138 patients who underwent FST, stratified by AEH and EC.

	Population	AEH	EC	*p*
	(*N* = 138)	(*N* = 79)	(*N* = 59)	
Age at diagnosis (year), median (IQR)	36.0 (31–40)	37.0 (32–40)	34.0 (29–38)	0.01
≥35, *n* (%)	78 (56.5%)	50 (63.3%)	28 (47.5%)	0.06
≥40, *n* (%)	36 (26.1%)	27 (34.2%)	9 (15.3%)	0.01
BMI (kg/m^2^), median (IQR)	25.1 (21.6–30.2)	23.4 (20.9–29.4)	25.7 (23.2–30.8)	0.03
≥25, *n* (%)	68/132 (51.5%)	32/75 (42.7%)	36/57 (63.2%)	0.02
≥30, *n* (%)	34/132 (25.8%)	18/75 (24.0%)	16/57 (28.1%)	0.60
Reproductive history				
Full term pregnancies, *n* (%)	18 (13.0%)	10 (12.7%)	8 (13.6%)	0.88
Miscarriages, *n* (%)	17 (12.3%)	9 (11.4%)	8 (13.6%)	0.70
Failed embryo transfer, *n* (%)	4 (2.9%)	1 (1.3%)	3 (5.1%)	0.21
Extension				0.04
Focal, *n* (%)	84 (60.9%)	54 (68.4%)	30 (50.8%)	
Diffuse, *n* (%)	54 (39.1%)	25 (31.6%)	29 (49.2%)	
Progestin therapy				<0.01
Only IUD, *n* (%)	87 (63.1%)	68 (86.1%)	19 (32.2%)	
Only Oral, *n* (%)	9 (6.5%)	5 (6.3%)	4 (6.8%)	
IUD + Oral, *n* (%)	42 (30.4%)	6 * (7.6%)	36 ** (61.0%)	

* 2 patients due to disease persistence and 4 due to relapse; none at baseline. ** all grade 2 cases (*n* = 24) at baseline; among grade 1 cases, 11 at baseline (according to caregiver’s decision) and 1 after relapse.

**Table 2:** Oncological outcomes of patients treated with FST, stratified by AEH and EC.

	AEH (*N* = 78)	EC (*N* = 56)	*p*
Complete response (CR)	94.9% (74/78)	98.2% (55 */56)	0.30
Time to CR (median, range)	6 (6–12)	6 (6–12)	0.42
Relapses	16.7% (13 **/78)	26.8% (15 ***/56)	0.16

* 1 patient partial response (downgraded to AEH); ** 8 patients after progestin therapy discontinuation for pregnancy attempt and 5 during ongoing treatment; *** 13 patients after progestin therapy discontinuation for pregnancy attempt and 2 during ongoing treatment (7 grade 1 and 8 grade 2; with 1 grade 1 relapsed as AEH).

**Table 3:** Reproductive outcomes of patients undergoing FST who attempted pregnancy, stratified by AEH and EC.

	AEH (*N* = 40)	EC (*N* = 31)
Clinical pregnancy rate (CPR)	47.5% (19/40)	54.8% (17/31)
Live birth rate (LBR)	57.9% (11 */19)	70.6% (12 **/17)
Miscarriage rate (MR)	15.8% (3/19)	17.6% (3/17)
Ongoing pregnancies	26.3% (5/19)	11.8% (2/17)
Attempted pregnancy with ART	30.0% (12/40)	25.8% (8/31)

* 7 vaginal deliveries and 4 cesarean sections; ** 7 vaginal deliveries and 5 cesarean sections.

**Table 4:** Multivariate binomial logistic regression analyses with CR at 6 months and disease relapse as dependent variables.

	Complete Response			Relapses		
	*p*	OR	OR (95% CI)	*p*	OR	OR (95% CI)
Age ≥ 35	0.03	0.37	0.15–0.90	0.01	3.80	1.34–10.74
BMI ≥ 25	0.54	1.30	0.56–3.02	0.15	1.99	0.77–5.15
EC (vs. AEH)	0.68	0.81	0.29–2.22	0.83	1.13	0.36–3.56
Diffuse extension (vs. focal)	0.62	0.81	0.35–1.87	0.55	1.33	0.52–3.35
Grade 2 (vs. Grade 1-AEH)	0.61	0.72	0.21–2.52	0.21	2.32	0.62–8.66

*p* value, odds ratio (OR) and its 95% confidence interval (CI) are reported for each variable.

## Data Availability

The data presented in this study are available from the corresponding author upon request.

## Abbreviations

The following abbreviations are used in this manuscript:

FST	Fertility-sparing treatment
AEH	Atypical endometrial hyperplasia
EC	Endometrial cancer
D&C	Dilation and curettage
TRD	Tissue removal device
LNG-IUD	Levonorgestrel-releasing intrauterine device
CR	Complete response
CPR	Clinical pregnancy rate
MR	Miscarriage rate
LBR	Live birth rate
ART	Assisted reproductive technology
BMI	Body mass index
MMR	Mismatch repair
ER	Estrogen receptor
PR	Progesterone receptor
